# Correction: Wang et al. Current and Potential Future Global Distribution of the Raisin Moth *Cadra figulilella* (Lepidoptera: Pyralidae) under Two Different Climate Change Scenarios. *Biology* 2023, *12*, 435

**DOI:** 10.3390/biology12081045

**Published:** 2023-07-25

**Authors:** Bing-Xin Wang, Liang Zhu, Gang Ma, Adriana Najar-Rodriguez, Jin-Ping Zhang, Feng Zhang, Gonzalo A. Avila, Chun-Sen Ma

**Affiliations:** 1 School of Life Science, Institutes of Life Science and Green Development, Hebei University, Baoding 071002, China; 2 Climate Change Biology Research Group, State Key Laboratory for Biology of Plant Diseases and Insect Pests, Institute of Plant Protection, Chinese Academy of Agricultural Sciences, Beijing 100193, China; 3 Wildlife Ecology and Conservation Group, Wageningen University & Research Centre, Droevendaalsesteeg 3a, 6708 PB Wageningen, The Netherlands; 4 The New Zealand Institute for Plant and Food Research, Canterbury Agriculture and Science Center, Lincoln 7608, New Zealand; 5 CABI East & South-East Asia, 12 Zhonggunancun Nandajie, Beijing 100081, China; 6 MARA-CABI Joint Laboratory for Biosafety, Institute of Plant Protection, Chinese Academy of Agricultural Sciences, Beijing 100193, China; 7 The New Zealand Institute for Plant and Food Research Limited, Auckland Mail Centre, Private Bag 92169, Auckland 1025, New Zealand

## 1. Table Legend

In the original publication [1], there was a mistake in the legend for Tables 1, 2 and A2. The correct legend appears below:

Table 1. CLIMEX parameter values used to fit the potential distribution of *Cadra figulilella*. Footnote: Values without units are dimensionless indices. Definitions of all CLIMEX parameters included in this table are described in Kriticos et al. [14].

Table 2. Percentage contribution and permutation importance values for each environmental variable included in a MaxEnt model used to predict current and future worldwide distributions of the raisin moth, *Cadra figulilella*.

Table A2. List of bioclimatic variables (bio1–bio19) and topographic variables (ELEV, ASPECT, SLOPE), available on the WorldClim website (https://www.worldclim.org/). The variables highlighted in bold were chosen to construct the MaxEnt model used in this study to predict areas that may be suitable for the development of the raisin moth under current and future climates.

## 2. Table Citation Correction

In the original publication, there was a typo in the last sentence of Section 2.1.2. The appropriate reference for the table in the main text is Table A2 rather than Table A1.

## 3. Error in Figure

In the original publication [1], the original versions of Figures 1–3 were mistakenly published as the final versions. Below are the correct versions of Figure 1, Figure 2 and Figure 3.

## 4. Error in Affiliation

In the published publication [1], there was an error regarding the affiliation for Gonzalo A. Avila. The original affiliation 8 should be deleted.

## 5. Error in Author Contributions

In the published publication [1], there was an error regarding the author contributions for Gonzalo A. Avila. Funding acquisition for this author should be deleted.

## 6. Text Correction

The author would like to make the following corrections to the published paper [1]:

Section 2.1.2, Paragraph 2. The last sentence “We chose two shared socioeconomic pathways scenarios (SSP1-2.6 and SSP5-8.5) for two target time periods (mid-century: 2041–2060 and the end of century: 2081–2100), which represented the “best” and the “worst” warming future. SSP1-2.6 represents a low-emissions scenario with a warming projection of 2 °C, and SSP5-8.5 represents high-emissions scenarios with a warming projection of 5 °C (relative to the years 1880–1900), respectively” should be replaced with “We chose two shared socioeconomic pathways scenarios (SSP1-2.6 and SSP5-8.5) for two target time periods (mid-century: 2041–2060 and the end of century: 2081–2100), which represented the “best” and the “worst” warming future. SSP1-2.6 represents a low-emission scenario with a warming projection of 2 °C, and SSP5-8.5 represents a high-emission scenario with a warming projection of 5 °C (relative to the years 1880–1900), respectively”.

Section 2.1.2, Paragraph 3. The text “These variables were selected on the basis of their potential biological relevance to the raisin moth and on their use in previous niche modelling studies of the insect pest [19]” should be replaced with “These variables were selected on the basis of their potential biological relevance to the rai-sin moth and on their use in previous niche modelling studies of the insect pests [19]”.

Section 2.2.2, Paragraph 1. The first sentence should be shorten as “MaxEnt uses presence-only data as an input to predict the potential distribution of a given species”. We acknowledge that our readers may be experts on the MaxEnt model and may find this sentence unnecessary.

Section 3.1.2, Paragraph 1. The text “In addition, avg.OR10 = 0.1595 was 61.83% lower than those of the MaxEnt model with the default settings (avg.OR10 = 0.4179)” should be replaced with “The average 10% training omission rate (avg.OR10) value obtained, which was 0.1595, was 61.83% lower than those of the MaxEnt model with the default settings (avg.OR10 = 0.4179)”.

Section 3.1.2, Paragraph 2. In the last sentence, the text “The probability that the raisin moth was present was high between the 18° and 45° latitude, while it was very low when the latitudes were <−50°” should be replaced with “The probability of the raisin moth being present was high between latitudes 18° and 45°, while it was very low at latitudes <−50°”.

Section 3.2.1. In the last paragraph, the text “The potential total suitable distribution areas was not expected to significantly change from current (4.70 × 10^7^ km^2^) to the end of this century (4.94 × 10^7^ km^2^) under SSP1-2.6” should be replaced with “The predicted global distribution areas under SSP1-2.6 climate change scenario (4.94 × 10^7^ km^2^) are not expected to change much from the global predicted distribution under current climate (4.7 × 10^7^ km^2^)”.

Section 3.3. The text “However, MaxEnt’s prediction of a larger increase in suitable regions can be attributed to CLIMEX’s larger predicted decrease in suitable regions. Additionally, MaxEnt may have overestimated due to its insufficient data regarding the thermal requirements of the raisin moth” should be replaced with “However, the MaxEnt model predicts a larger number of suitable areas for the establishment of the raisin moths than CLIMEX predictions. Additionally, predictions in some areas, such as South America, are rather different”.

Section 4, Paragraph 6. In the first sentence, “with the exception of CLIMEX that also can include biological data of the species and non-climatic factors (like irrigation)” should be added at the end of the sentence.

Section 4. In the last paragraph, the text “However, in the current scenarios, the substantial discrepancy in predictions of climate change impacts between the models only serves to compound the uncertainty, as it is challenging to determine which one is more reliable” should be replaced with “However, in the current scenarios, the substantial discrepancy in predictions of climate change impacts between the models (and also predictions in a number of areas (e.g., South America)), under the current climate, only serves to compound the uncertainty, as it is challenging to determine which one is more reliable”.

The authors state that the scientific conclusions are unaffected. This correction was approved by the Academic Editor. The original publication has also been updated.

## Figures and Tables

**Figure 1:**
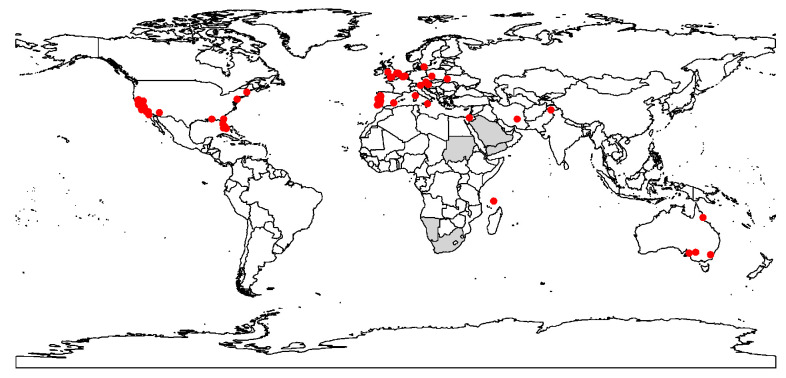
Current worldwide distribution (red dots) of the raisin moth, *Cadra figulilella*. Grey areas represent the distribution of the raisin moth from https://www.afromoths.net/species/show/11662 (accessed on 3 March 2023). These areas were excluded in the model fitting process due to insufficient spatial data (i.e., their precise geographic coordinates could not be determined).

**Figure 2:**
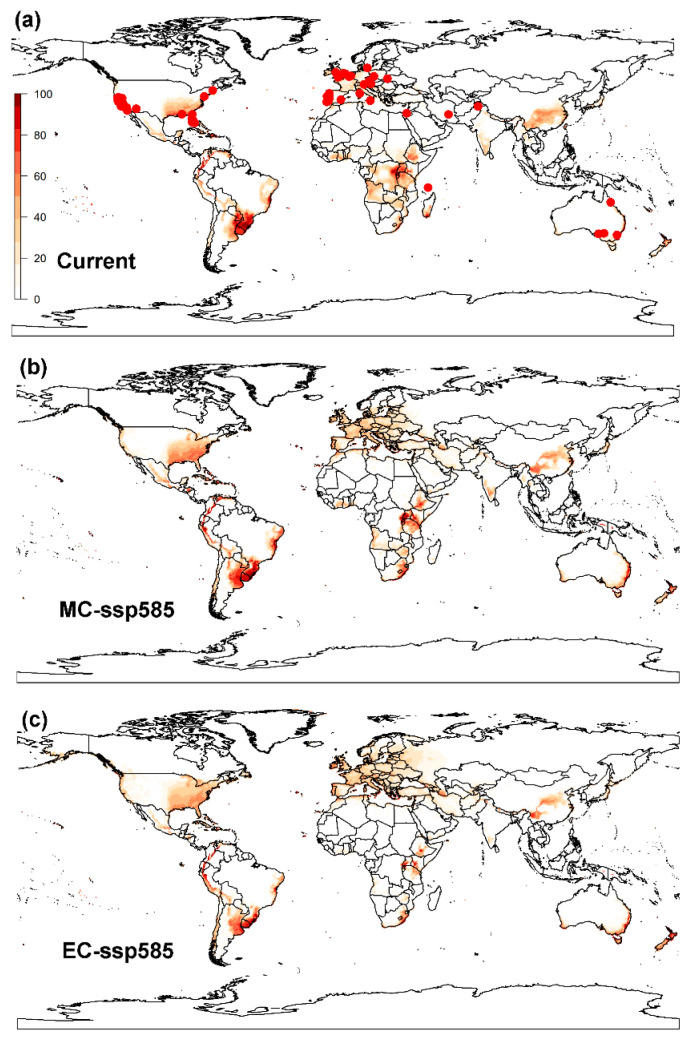
Global potential distribution of the raisin moth, *Cadra figulilella*, predicted by CLIMEX, where (**a**) denotes the potential distribution under current climate conditions, (**b**) denotes the potential distribution under the ssp585 scenarios by mid-century (MC, 2041–2060), (**c**) denotes the potential distribution under the ssp585 scenarios by end of century (EC, 2081–2100). Dark red areas (EI = 100) denote highly suitable areas, while white areas represent unsuitable areas (EI = 0). Red dots denote the current worldwide distribution of the raisin moth.

**Figure 3:**
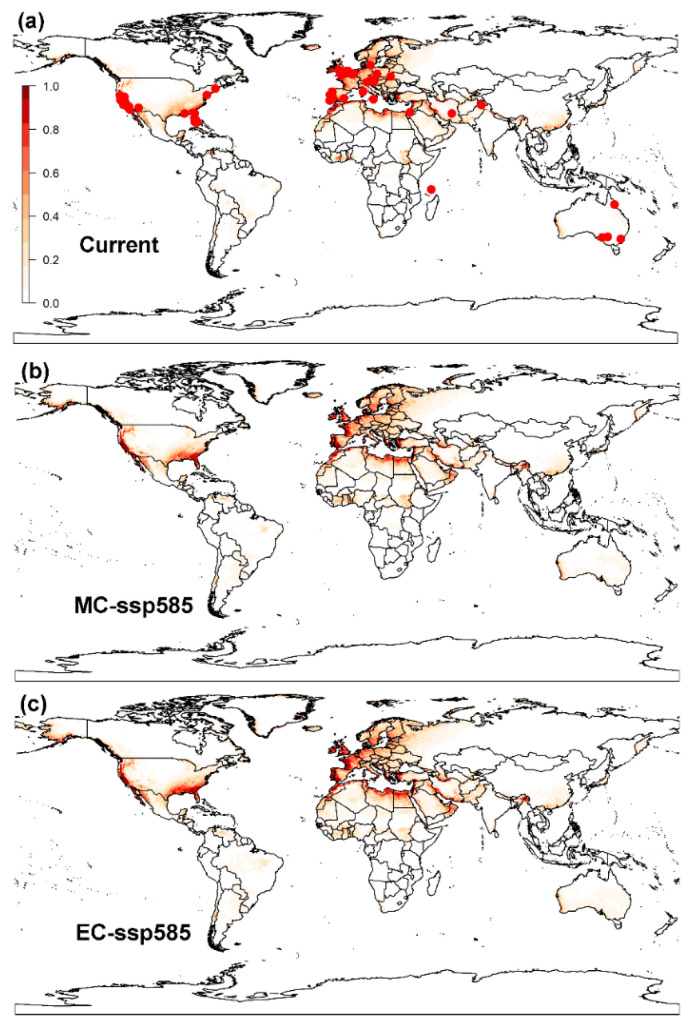
Global potential distribution of the raisin moth, *Cadra figulilella*, predicted by MaxEnt, where (**a**) denotes the potential distribution under current climate conditions, (**b**) denotes the potential distribution under the ssp585 scenarios by mid-century (MC, 2041–2060), (**c**) denotes the potential distribution under the ssp585 scenarios by end of century (EC, 2081–2100). Dark red areas denote highly suitable areas, while white areas represent unsuitable areas. Red dots denote the current worldwide distribution of the raisin moth.
